# Strategies for Using Muramyl Peptides - Modulators of Innate Immunity of Bacterial Origin - in Medicine

**DOI:** 10.3389/fimmu.2021.607178

**Published:** 2021-04-20

**Authors:** Svetlana V. Guryanova, Rahim M. Khaitov

**Affiliations:** ^1^ Shemyakin–Ovchinnikov Institute of Bioorganic Chemistry, Russian Academy of Sciences (RAS), Moscow, Russia; ^2^ Department of Biology and General Genetics, Medical Institute, RUDN University, Moscow, Russia; ^3^ National Research Center – Institute of Immunology of Federal Medico-Biological Agency, Moscow, Russia; ^4^ Department of Immunology, Moscow State University of Medicine and Dentistry, Moscow, Russia

**Keywords:** innate immunity, muramyl peptides, glucosaminylmuramyldipeptide, treatment, disease prevention, microflora, infection, NOD2 receptors

## Abstract

The spread of infectious diseases is rampant. The emergence of new infections, the irrational use of antibiotics in medicine and their widespread use in agriculture contribute to the emergence of microorganisms that are resistant to antimicrobial drugs. By 2050, mortality from antibiotic-resistant strains of bacteria is projected to increase up to 10 million people per year, which will exceed mortality from cancer. Mutations in bacteria and viruses are occurring faster than new drugs and vaccines are being introduced to the market. In search of effective protection against infections, new strategies and approaches are being developed, one of which is the use of innate immunity activators in combination with etiotropic chemotherapy drugs. Muramyl peptides, which are part of peptidoglycan of cell walls of all known bacteria, regularly formed in the body during the breakdown of microflora and considered to be natural regulators of immunity. Their interaction with intracellular receptors launches a sequence of processes that ultimately leads to the increased expression of genes of MHC molecules, pro-inflammatory mediators, cytokines and their soluble and membrane-associated receptors. As a result, all subpopulations of immunocompetent cells are activated: macrophages and dendritic cells, neutrophils, T-, B- lymphocytes and natural killer cells for an adequate response to foreign or transformed antigens, manifested both in the regulation of the inflammatory response and in providing immunological tolerance. Muramyl peptides take part in the process of hematopoiesis, stimulating production of colony-stimulating factors, which is the basis for their use in the treatment of oncological diseases. In this review we highlight clinical trials of drugs based on muramyl peptides, as well as clinical efficacy of drugs mifamurtide, lycopid, liasten and polimuramil. Such a multifactorial effect of muramyl peptides and a well-known mechanism of activity make them promising drugs in the treatment and preventing of infectious, allergic and oncological diseases, and in the composition of vaccines.

## Introduction

Over the past decades, impressive advances have been made in the treatment and prevention of many dangerous infections. However, the achievements of medicine of the last century may be lost due to the emergence and spread of deadly infectious diseases. Previously defeated viral and bacterial infections can become incurable and spread throughout the world (1).

In the United States alone, in 14 months, the Coronavirus disease (COVID-19) pandemic claimed the lives of 500 thousand people (2). Every year more than 2.8 million people are infected with antibiotic-resistant strains and 35 thousand die as a result (3). In the European Community, there have been more than 900,000 deaths from COVID-19 in the 14 months since the first recorded European death on February 15 (4), and there are 2.5 million infected with antibiotic-resistant strains resulting in 25,000 deaths annually (5). In 2013, the professional medical community recognized the onset of the post-antibiotic era (6), and in 2014 the World Health Organization issued a report in which it warned of dire consequences: the most common infections can become fatal due to the increase in the virulence of microorganisms and the emergence of strains resistant to all known antibiotics (7). Scientists predict that by 2050 the death rate from antibiotic-resistant strains of bacteria will be 10 million people a year, and will exceed the death rate from cancer (8).

Tremendous efforts of the largest pharmaceutical companies are aimed at finding new antiviral drugs and antibiotics, but as soon as they appear, viruses and bacteria find a way to resist them. It must be admitted that mutations in microorganisms occur faster than new antimicrobial drugs are introduced to the market. Over the past 10 years, 15 antibiotics have been developed, of which only 3 have passed all phases of testing and have been approved for medical use (5). In search of effective protection against infections, new strategies and approaches are being developed, one of which is immunomodulatory therapy. Immunomodulatory therapy in combination with etiotropic chemotherapy is increasingly being used in the treatment and prevention of diseases of various nosological forms. It is not surprising that the sales of immunotropic drugs are growing at an outstanding pace: the growth in the sales of immunomodulators in Russia in March 2020 amounted to 50.9% (9); the volume of the pharmaceutical market of immunomodulators and anticancer drugs in Russia in 2020 reached 36,4 billion rubles (9), in the USA $ 60.4 billion and 157.34 billion dollars in the world (10). The world market of immunomodulators is one of the most dynamic segments of the pharmaceutical market: the average annual growth rate in the next 10 years is expected to be 5.4%, the volume in 2027 - USD 251.69 Billion (10).

Nowadays, immunomodulators include drugs based on antibodies, receptors, cytokines, nucleic acids, peptides, drugs of bacterial and plant origin, synthetic drugs. Immunomodulators of bacterial origin and their semisynthetic analogs have a longer history of use than synthetic ones, and in recent years have become one of the promising directions. This is due to fundamental revolutionary discoveries in the field of immunology, the understanding of the principles of innate immunity, based on the recognition of Pathogen-associated molecular patterns (PAMPs) of foreign organisms. Muramyl peptides are bacterial PAMPs as far as they are fragment of peptidoglycan (or murein) of all known bacteria. Peptidoglycan is located outside the plasma membrane of bacteria and form bacterial cell wall. It performs supporting and protective functions. In different bacterial species, additional residues of fatty acids, peptides, and carbohydrates can be found in the connection of peptide chain of the peptidoglycan. Peptidoglycan undergoes structural rearrangements in the process of bacterial growth and division. These autolytic functions are performed by intracellular peptidoglycan hydrolases and amidases, which destroy peptidoglycan and form muramyl peptides (11–13).

In humans and animals, muramyl peptides appear under the action of host enzymes that break down bacterial peptidoglycans (14), as well as under the action of bacterial enzymes that are necessary for bacteria to remodel cell wall peptidoglycan during division and growth (15–17) and during competition of bacteria in the conquest of new niches (18–20). Muramyl peptides can influence the developmental cycle of bacteria, in particular, anhydro-GMDP promotes the exit from the dormant form of mycobacteria (21).

MDP analogs which are present in the body of animals and humans, affect the nervous (22) and immune system (23–25), they are involved in the regulation of host metabolism (26). The wide range of immunological activities gave a reason to consider muramyl peptides “vitamins of the immune system” (23, 27).

Muramyl peptides, according to numerous studies, are the minimal biologically active fragments that initiate immune response after interacting with intracellular receptors of innate immunity NLR family (NOD-Like Receptors - NOD1, NOD2, NALP3, etc.) (28–30). The interaction of muramyl peptides with NLRs triggers a signaling cascade of reactions that induces the expression of a large amount of genes, in particular, genes of pro-inflammatory cytokines (interleukins IL-1, IL-2, IL-6, IL-8, IL-12, tumor necrosis factor-alpha, interferons), adhesion molecules, acute phase proteins, inflammation enzymes (NO synthase and cyclooxygenase), molecules of the main histocompatibility complex, colony stimulating factors (CSF) (23, 31, 32). The result of the activation of NLR receptors is an inflammatory response of the body, manifested in the enhancement of the antimicrobial function of neutrophils (33–35), monocytes and macrophages (36, 37), an increase in the cytotoxic activity of natural killer cells (NK cells) (38, 39), activation of B- cells (40), CD8 +, γδ T cells (41–43), which ultimately leads to the elimination of the pathogen. Induction of CSF synthesis activates leukopoiesis, correcting cytopenias (44–46), and leads to the emergence of immunological tolerance to foreign antigens, including commensals (47). Muramyl peptides, by activating the NOD2 receptor, promote synergistic cooperation of intracellular NF-kB-MAPK- and IRF-signaling cascades under the joint action of TLR4 and TLR9 agonists (48).

It was shown that the activation of the NOD2 receptor is a requirement for adaptive immunity to bacterial (49, 50), viral (42), parasitic (51) infections, contributing to the formation of trained immunity - long-term epigenetic changes in cells, which leads to their enhanced response to a secondary stimuli (52, 53).

Thus, muramyl peptides are involved in the stimulation of all forms of anti-infectious defense processes of the body: phagocytosis, cellular and humoral immunity, and they also take part in ensuring immunological tolerance and hematopoiesis, regulating immune homeostasis. Moreover, recent studies have shown the effect of muramyl peptides on bone metabolism and an increase in mineral density by enhancing osteoblast differentiation and expression of bone-forming genes. It is assumed that muramyl peptides are the new inducers of bone formation and can be used for protection against osteoporosis (54).

Numerous experiments have proven that NLRs are the main sensors for muramyl peptides, but recent data indicates that not only NLR structures bind to muramyl peptides. Members of the family of carriers of proton-linked oligopeptides (POTs) bind to and deliver muramyl peptides to the cytosol of the cell from the extracellular space and endosomes (37, 55, 56). Protein YB1 also interacts with muramyl peptides and causes the expression of the transcription factor NF-κB in the absence of NOD2 (24). Rat liver mitochondria isolated from cells reacts with muramyl peptide. It is noteworthy that MDP and MDP-Lys caused a significant decrease in respiratory control, reducing the efficiency of oxidative phosphorylation of mitochondria, while other muramyl peptides, both biologically active (murabutide) and inactive analogs (MDP-DD) did not affect the bioenergetics of mitochondria (57). Thus, the effect of muramyl peptides on the bioenergetic processes of mitochondria, independent of the activation of the NOD2 receptor, was demonstrated.

## The History of Muramyl Peptides Discovery

**Figure 1:**
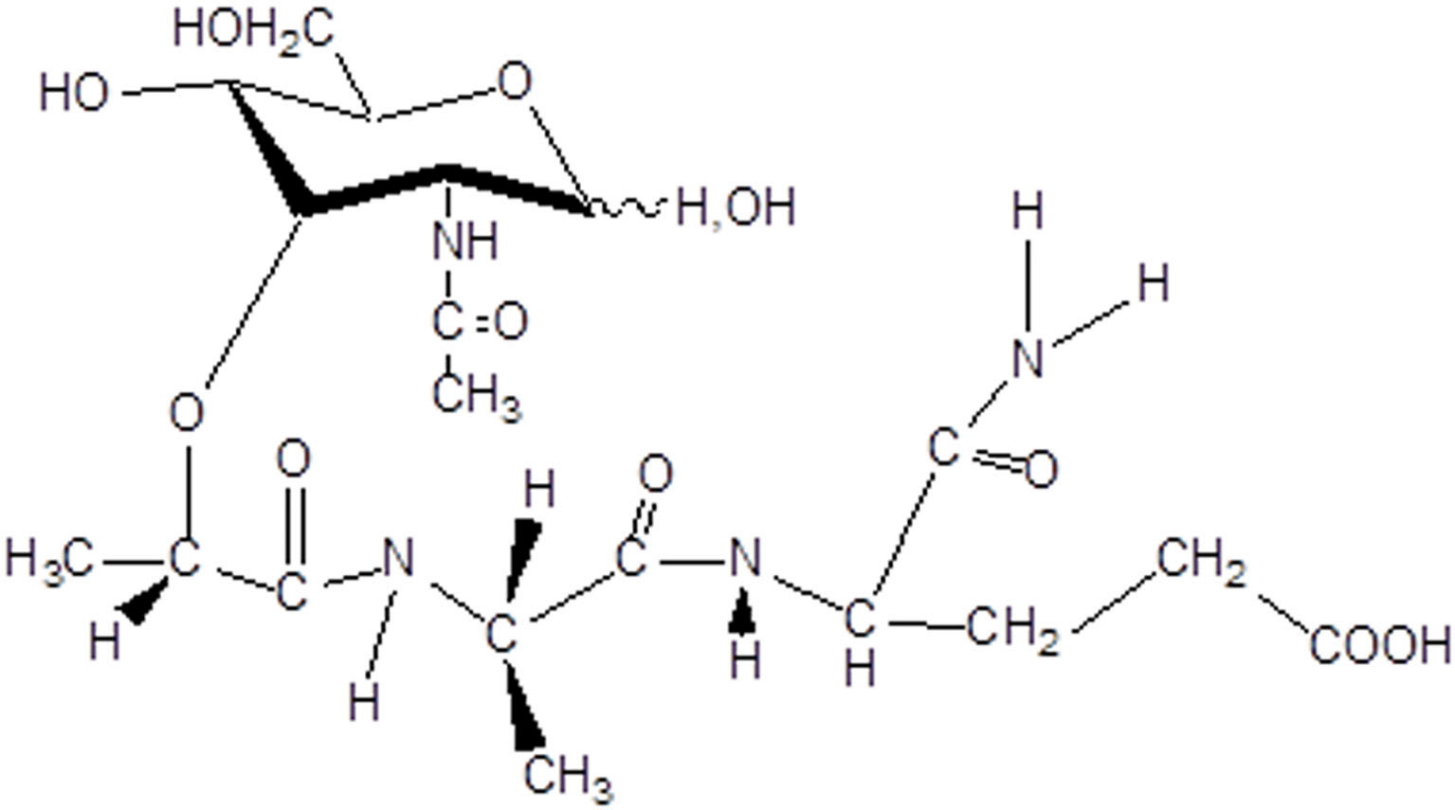
Structure of N-acetylmuramyl-L-alanyl-D-isoglutamine (MDP).

For more than fifty years, intensive research for biologically active derivatives of muramyl dipeptide (N-acetylmuramyl-L-alanyl-D-isoglutamine, MDP, **Figure 1**) has been carried out.

Scientific laboratories in Japan (58–60), France (61, 62) and Russia (63, 64), independently of each other, discovered the immunoadjuvant properties of muramyl peptides. In Japan, during investigation of the virulence of human tubercle bacilli, hundreds of compounds isolated from these microorganisms were tested, and it was found that glycopeptides of the cell walls of mycobacteria are responsible for the manifestation of biological and immunological activity (65–68). In France, the object of the study was Freund’s complete adjuvant containing killed bacteria *Mycobacterium tuberculosis*, and a BCG vaccine prepared from a strain of weakened live bovine tuberculosis bacillus which was used for the treatment of oncological diseases (69–71). It was shown that the minimal structural component responsible for the manifestation of adjuvant and immunological properties of whole mycobacterium, was a fragment of the cell walls N-acetylmuramyl-L-alanyl-D-isoglutamine (72).

**Figure 2:**
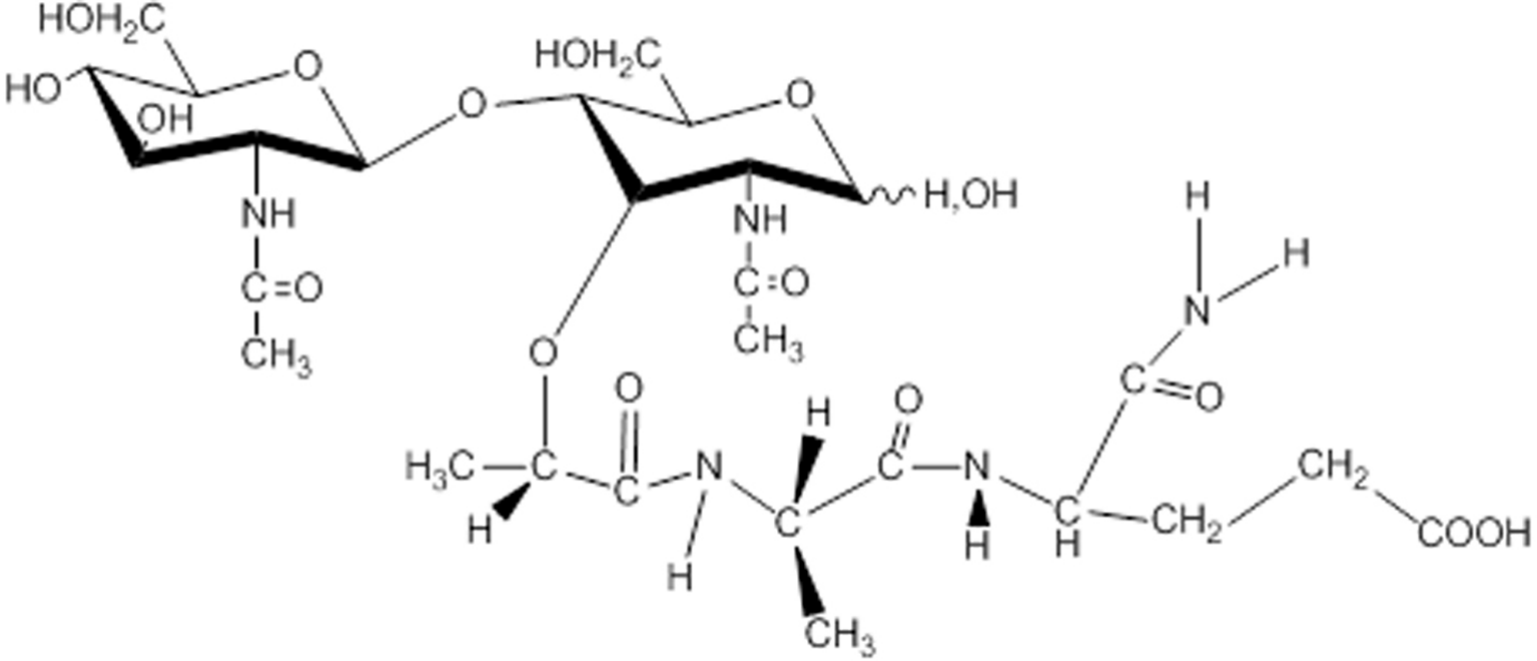
Structure of N-acetylglucosaminyl-N-acetylmuramyl-L-alanyl-D-isoglutamine (GMDP).

In Russia, studies of muramyl peptides began with the determination of the active substance of the lactic acid bacterium *Lactobacillus bulgaricus* (73), which performed anticancer and immunostimulating activity. At the Institute of Bioorganic Chemistry Russian Academy of Sciences (IBCh RAS) it was found that glycopeptide from *Lactobacillus bulgaricus* was N-acetylglucosaminyl-N-acetylmuramyl-L-alanyl-D-isoglutamine (GMDP, **Figure 2**). GMDP differs from MDP by the presence of N-acetylglucosaminyl (63). An original method for the synthesis of GMDP was developed in the laboratory of peptide chemistry at the IBCh RAS (64).

Subsequent numerous studies have shown that GMDP, like MDP, is a ligand of the NOD2 receptor (74), has a more adjuvanticity than MDP (75). GMDP stimulates anti-infectious resistance and antitumor immunity, activates immunocompetent cells, and induces synthesis of a number of cytokines and mediators inflammatory processes (76–78).

In addition, GMDP has a less pyrogenic effect than MDP. These properties of GMDP made it possible to create a drug on its basis. At the same time, the high pyrogenic activity of MDP served as an obstacle to its clinical use. There was a great number of studies examining the immunogenic and pyrogenic activities of muramyl peptides aimed to find the structural analogs, devoid of undesirable side effects (79).

To date, a great number of muramyl peptide derivatives have been synthesized. Some of them have passed clinical trials and are already being used in 40 countries (**Table 1**).

**Table 1:** Muramyl peptides registered and investigated as medicinal products.

Trade name INN Company	Chemical Names	Applying	Country/Date of registration
**Nopia®/**Romurtide, Muroctasin, *Daiichi Pharmaceutic*	N-Acetylmuramyl-Ala-D-isoglutaminyl-Nϵ-stearoyl-Lys,DJ-7041, MDP-Lys(L18)	Oncology, Neutropenia	Japan/1991
**B30-MDP** *Daiichi Pharmaceutic*	6-O-(2-tetradecylhexadecanoyl)-N-acetylmuramoyl-L-alanyl-D-isoglutamine)	Infectious diseases (adjuvant in influenza vaccine)	Japan/1992
**Licopid^®^** *AO Peptek*,	N-acetylglucosaminyl-N-acetylmuramyl-L-alanyl-D-isoglutamine, GMDP	Chronic recurrent respiratory tract infections, inflammatory diseases of skin and soft tissues, psoriasis, herpetic infections	Russia/1995; Belarus, Kazakhstan/2001; Azerbaijan, Armenia, Moldova/2007; Uzbekistan, Kyrgyzstan, Mongolia, Georgia/2015
**Threonyl Muramyl Dipeptide** *BOC Sciences*	Threonyl-N-Acetylmuramyl-Ala-D-isoglutamine Threonyl-MDP	HIV infection (adjuvant in AIDS vaccine)	USA, phase I/1996 The release of the medicinal product is suspended
**ImmTher^®^** *Immunotherapeutics Inc.*	N-acetylglucosamin-N- acetylmuramyl-L-alanyl-D- isoglutamine -L- alanyl- glyceryl dipalmitate, disaccharide tripeptide glycerol dipalmitoyl	Oncology	USA/2000
**Liasten^®^ (Blasten)** *Technologist*,	glucosaminylmuramyl pentapeptide GMPP	Infectious diseases, pulmonology, oncology, surgery	Ukraine/2000
**Murabutide** *ISTAK Biotechnology*,	N-acetylmuramyl-L-alanyl-D-isoglutamine-n-butyl ether	N-acetylmuramyl-L-alanyl-D-isoglutamine-n-butyl ether Infectious diseases, HIV infection	France/2002
**Mepact^®^,** Junovan, Mifamurtide *Takeda Pharmaceutical*	muramyltripeptide phosphatidyleanolamine incorporated into liposomes Liposomal Muramyltripeptide Phosphatidylethanolamine L-MTP-PE, MLV 19835A, MTP-PE	Oncology (osteosarcoma), adjuvant in the vaccine against AIDS and hepatitis B	27 countries of the European Union/2009 Mexico/2010
**norMDP, Almurtide^®^** *American Custom Chemicals Corp*	N-acetyl-nor-muramyl-L-alanyl-D-isoglutamine,Cgp-11637, Nor-MDP,UNII-1DCO35D4OR	Oncology (adjuvant in vaccine)	USA/1st phase 2011-2017
**Polimuramil^®^** *“Combotech” for LLC “CORUS PHARM”*	A complex of three components: 1) N-acetyl-D-glucosaminyl-N-acetyl-D-muramoyl-L-alanyl-D-isoglutaminyl-meso-diaminopimeli- new acid (HMtri); 2) N-acetyl-D-glucosaminyl-N-acetyl-D-muramoyl-L-alanyl-D-isoglutaminyl-meso-diaminopimeloyl-D-alanine (HMtetra); 3) dimer (HMtetra and diHMtetra)	Secondary immunodeficiency states, acute and chronic pyoderma complicated by secondary infection, dermatoses, treatment and prevention of surgical infections	Russia/2013

## Monosaccharide-Containing Muramyl Peptides

The first drug based on muramyl peptide N-acetylmuramyl-L-alanyl-D-isoglutamine-N6-steroyl-L-lysine, introduced into clinical practice, - **Nopia^®^** (**romurtide, muroctasine**) was chosen by Japanese scientists from several dozen of MDP derivatives, was able to increase the nonspecific resistance of animals to bacterial and viral pathogens (80). The ability of romurtide to induce cytokine synthesis was studied in details: IL-6, GM CSF and IL-1 were increased under its influence (81–83). This was the reason for studying its effect on leukopoiesis in cancer patients after chemotherapy or radiotherapy. The drug was injected subcutaneously the next day after the injection of chemotherapy drugs for 6 days at doses of 50 and 100 μg (84), 100 and 200 μg (85), 200 and 400 μg (86). As a result of clinical trials, it was found that the use of romurtide contributed to the rapid restoration of the number of leukocytes, mainly due to neutrophils. At the same time, a dose-dependent effect was observed: romurtide in a higher dosage was more effective in each of the studies, and at doses of 100 and 200 μg increased the number of neutrophils not only in blood, but also in the bone marrow. Stimulation of leukopoiesis in patients with solid tumors was higher than in patients with hematological malignant tumors, while a dosage of 400 μg caused side effects - fever, pain and redness at the injection site, headache; in this connection, the optimal dosage regimen was chosen to be 200 μg/day within 6 days. Further clinical studies of romurtide in the correction of side effects of anticancer chemotherapy and radiation therapy showed that it is effective not only in restoring the number of leukocytes, but also platelets, and intravenous administration is more preferable than subcutaneous administration (87). The data obtained allowed the conclusion that romurtide is a highly effective drug for restoring the number of leukocytes and platelets in cancer patients after chemotherapy or radiotherapy (88). Romurtide is the first muramyl dipeptide-derived immunotherapeutic drug approved for medical use in leukopenia. Later in 2011, the potentiating effect of romurtide was discovered: its co-administration with IFN-β promotes the maturation of dendritic cells and inhibits the growth of B16F10 melanoma, while muramyl peptide and IFN-β alone did not have a significant effect on tumor growth (89). In addition, through animal experiments, the Japanese researchers found the analgesic effect of romurtide: at doses of 10, 50, and 2.0 µg per mouse, a decrease in the frequency of convulsive movements caused by acetic acid was observed (90). Until 1998, the dosage form (DF) of romurtide was produced in the form of a lyophilisate of 200 μg in vials complete with water for preparing a solution for subcutaneous injection. Currently, the release of romurtide DF has been suspended, the substance is produced by several companies, including Invivogen (San Diego, CA, USA) and Ning Zhang Shaanxi Dideu Medichem Co. Ltd. (Shaanxi, China). Romurtide is included in the compositions of several thousand patents, most of which are registered by Amgen Inc.

One of the directions in the creation of immunotherapeutic drugs of muramyl dipeptide nature is the development of lipophilic derivatives. In these cases, fatty acids or phospholipids are attached to muramyl peptide. The first lipophilic analogue of muramyl peptides that passed the 1st phase of clinical trials is 6-O- (tetradecylhexadecanoyl) -N-acetylmuramyl-L-alanyl-D-isoglutamine (**B3O-MDP**) (91). Volunteers (N = 77) were divided into several groups, each of them was injected subcutaneously once or twice with a 4 week interval into the forearm area with a liposomal vaccine containing hemagglutinin and influenza virus neuraminidase with or without muramyl peptide. The inclusion of muramyl peptide B30-MDP as an adjuvant in the composition of an influenza vaccine containing hemagglutenin and neuraminidase of the influenza virus induces 16 times stronger antibody production compared to the vaccine without muramyl peptide. An incorporation vaccine component into cholesterol particles (virosomes) lengthens antibody production up to 6 months (92). Local adverse reactions observed with the introduction of MDP virosome vaccines in the form of redness, swelling, and pain at the injection site, disappeared within 5 days. Systemic adverse reactions were manifested in the form of leukocytosis. Currently, the B30-MDP dosage form is not produced, but is included in the composition of numerous European and American patents as an adjuvant, for example, patents WO2017023782A1 (2017), US20150306198A1 (2015), WO2011085071A2 (2011), US20130202655A1 (2011), WO2008070564A1 (2007).

The muramyl peptides **Threonyl-MDP** and **MTP-PE** were used as an adjuvant in an AIDS vaccine in a randomized, blinded, placebo-controlled phase I clinical trial. In 112 healthy HIV-negative volunteers, the ability of the rgp 120/SF-2 vaccine to induce a delayed-type hypersensitivity reaction when administered subcutaneously in the presence of adjuvants was determined (93, 94). Due to limited funding, further research was discontinued (95). Threonyl-MDP is part of the composition of more than 4 thousand patents, most of which are registered by Novartis International Ag.

Muramyltripeptide phosphatidylethanolamine (**MTP-PE, Mifamurtide**) incorporated into liposomes (L-MTP-PE, trade name **Mepact)** is registered in 27 EU countries as adjuvant therapy in combination with traditional chemotherapy in the treatment of osteosarcoma (96, 97), an orphan disease affecting mainly children and young people with a 30% fatal outcome. In preclinical studies on dogs with osteosarcoma, it was shown that intravenous administration of L-MTP-PE in the postoperative period increased the life expectancy of animals threefold (222 days versus 77 days). Moreover, in the group of 14 animals receiving L-MTP-PE, 4 of them were completely cured. The drug was well tolerated, and there were no toxic effects, with a moderate increase in body temperature (by 1-2°C) within 2-6 hours after injection (98). Phase I clinical trials have confirmed the safety of mifamurtide when administered intravenously at a dose of 2 mg twice a week for 12 weeks and then for 24 weeks once a week (99). As a result of the phase I trials, the maximum tolerated dose was not reached, therefore a biologically optimized dose of 2 mg was chosen for the phase II trials. Phase II studies have shown a reduction in the risk of further relapse and a twofold increase in remission in patients with inoperable tumors who received mifamurtide in combination with chemotherapy (100). A phase III randomized trial demonstrated a 30% reduction in the risk of death from osteosarcoma, which allowed the European Medicines Agency (EMA) to recommend the inclusion of mifamurtide in the systemic therapy of metastatic osteosarcoma (101). It is known that metastasis in the lungs is the main aggravating factor in the prognosis of osteosarcoma. In an experimental model, it was shown that the inclusion of mifamurtide in a complex therapy at a dosage of 1 mg/kg eliminates metastasis in the lungs and increases the response to chemotherapy (102). Impressive results were also obtained when mifamurtide was included as an adjuvant in the vaccine against hepatitis B: immunogenicity increased up to 4-5 times, and the level of IFN-gamma increased (103). Mifamurtide is a part of the composition of more than 500 patents, most of which are registered by Bayer Pharma Ag.

**Murabutide**, a pyrogen-free MDP (N-acetylmuramyl-L-alanyl-D-isoglutamine-N-butyl ether, ISTAC Biotechnology, Lille, France) is a safe derivative of MDP. Macrophages stimulated by murabutide increased expression of genes encoding various proteins, such as immune mediators and their receptors, transcription factors and kinases, transporters and proteins involved in the metabolic activity of cells, reflecting a wide range of biological effects. Murabutide also enhances host resistance to microbial infections, nonspecific resistance to tumors, and induction of cytokines and chemokines involved in enhancing the immune response and hematopoiesis (104). Murabutide passed the 1st and 2nd phases of clinical trials in France; with practically no side effects. 6-8 hours after the subcutaneous injection of 100 μg/kg murabutide to 12 healthy men the level of neutrophils was significantly increased, which returned to normal after 24 hours. Only 2 cytokines were detected in blood serum using the enzyme immunoassay: IL-6 and granulocyte colony-stimulating factor (GCSF). At the same time, a certain amount of soluble receptors for TNF and IL-1, which are known to be antagonists of these proinflammatory cytokines, were found in serum of all examined patients (105). The effect of murabutide on cytokine production was analyzed in human whole blood. After 2 hours, an increase in the level of cytokines, such as tumor necrosis factor (TNF), interleukin-1 beta (IL-1 beta), IL-6, IL-8, as well as the anti-inflammatory mediator IL-1Ra, was recorded. Studies of action of murabutide with cytokines have shown that in combination with TNF, the secretion of IL-6 by human monocytes increases, with murabutide in combination with IL-2 or IL-4, the proliferation and differentiation of B-lymphocytes was detected. The combination of interferon-alpha-2a (1 million units) and murabutide (7 mg) induced a significant increase in the level of CSF, IL-6, soluble receptors for IL-1 and TNF in the blood. Experimental results have shown that the joint introduction of murabutide with cytokines can have a synergistic effect, thus decreasing the effective doses of the recombinant cytokine.

It is known that the correction of the immunity of HIV-infected patients contributes to enhanced restoration of immunity and effective control of virus replication. Murabutide regulates the function of antigen-presenting cells and selectively activates CD4 lymphocytes, which leads to a suppression of HIV replication *in vitro*. Therefore, phases 1 and 2 of clinical trials were focused on the safety and efficacy of murabutide in HIV-infected patients receiving antiretroviral therapy. The first study showed that a single dose of 5, 7, or 9 mg of murabutide (6 patients per dose) was well tolerated. Selective induction of serum cytokines and chemokines was observed, with cytokine levels reaching a plateau at a dose of 7 mg. The second study evaluated the safety and biological effects of repeated administrations of 7 mg murabutide, for 5 consecutive days, to 12 HIV-1-infected patients. During the study, good tolerability was noted. Moreover, the retention of the clinical effect was observed for 3 weeks after the administration of murabutide (106). When studying the ability of murabutide to control the replication of human immunodeficiency virus type 1 (HIV-1) in infected monocytes and dendritic cells, significant suppression of viral replication in both types of cells was found. Murabutide did not affect the penetration of the virus into the cell, the activity of reverse transcriptase, or the early formation of proviral DNA in the cytoplasm of infected cells. However, a drastic reduction in viral mRNA was observed in monocytes and in dendritic cells. This HIV-1 inhibitory activity was not mediated by inhibition of cellular DNA synthesis or activation of p38 mitogen-activated protein kinase. In addition, murabutide-stimulated cells expressed reduced amounts of receptors CD4 and CCR5 and secreted high levels of beta-chemokines. Neutralization of the released chemokines did not alter the HIV-1 inhibitory activity of murabutide. These results indicate that the immunomodulator murabutide used in the clinic can activate multiple effector pathways, and, as a consequence, inhibition of HIV-1 replication occurs (107). Murabutide is a part of the composition of about 200 patents, most of which are registered by Virochem Pharma Inc.

Muramylpeptide **norMDP (Almurtide^®^)** is currently in phase 1 clinical trials as an adjuvant in the HER-2 vaccine against stomach, breast, and ovarian cancer (108). HER-2 (CD340) - a membrane protein, a tyrosine protein kinase of the epidermal growth factor receptor family, whose overexpression plays an important role in the pathogenesis and progression of certain aggressive types of cancer, is an important biomarker and therapeutic target of this disease (109). The first phase of an open, non-randomized clinical trial of a vaccine containing peptides (597-626) and (266-296) of the HER-2 protein, emulsified using norMDP in Montanide ISA 720 adjuvant, has been successfully completed (110, 111). The study design included intramuscular administration of three injections (every 21 days) of the vaccine in a final volume of 1.0 ml. First cohort received 1.0 mg of each peptide for vaccination; the next cohorts received 1.5 mg, 2.0 mg and 2.5 mg. The norMDP content in each injection was 0.025 mg. After completion of the course of treatment, patients were followed up for 30 days. A total of 24 patients participated in the study, 6 per group; as the optimal biological dose, a dosage of 1.5 mg of each peptide was chosen. The next stage 2 with the inclusion of 12 patients will end in December 2021. To assess the effectiveness of the therapeutic effect of the combination of HER-2 epitopes with adjuvants norMDP and Montanide ISA 720, it is planned to evaluate both humoral and cellular immunity, as well as pharmacokinetics and the mechanism of action (108). norMDP is a part of the composition of about 200 patents, most of which are registered by Novartis International Ag.

## Disaccharide-Containing Muramyl Peptides

**Figure 3:**
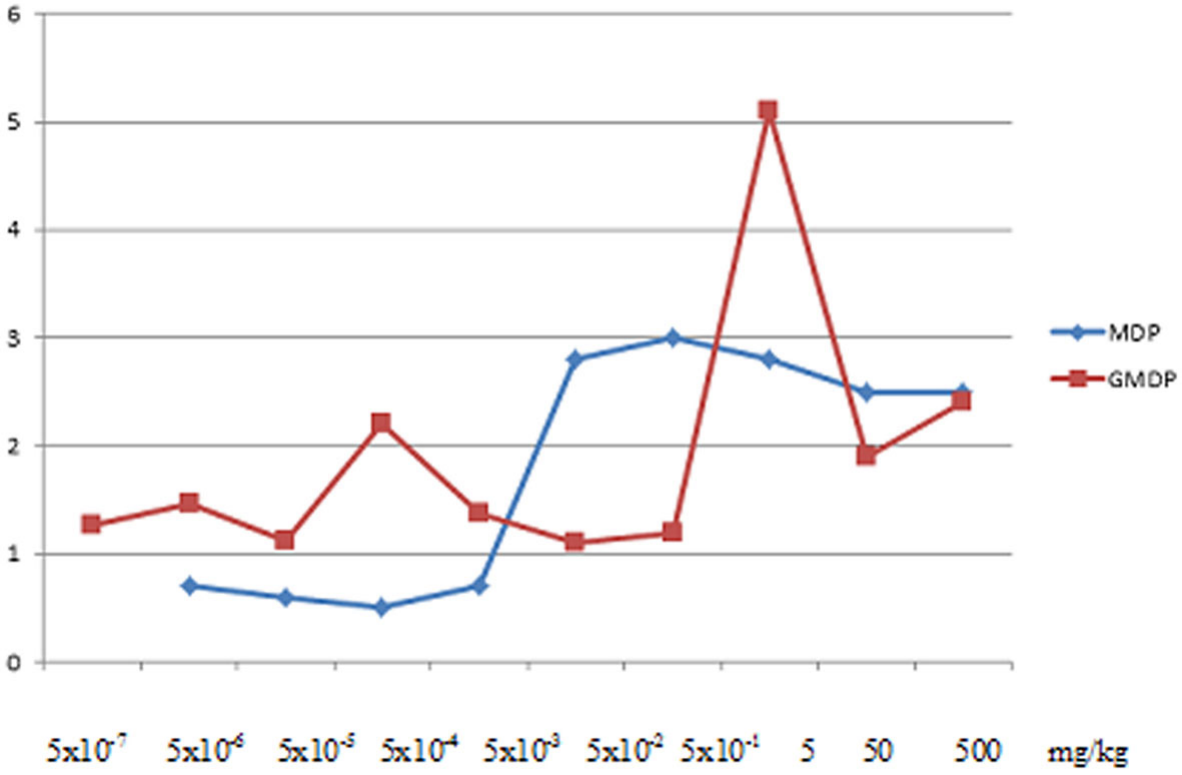
Indices of stimulation of the humoral response to ovalbumin in BALB/C mice after intraperitoneal administration of MDP and GMDP in the range from 5x10^-7^ to 5x10^2^ mg/kg (6 animals per dose), p < 0.05 (79).

Disaccharide-containing muramyl peptide derivatives are widely used in medical practice as immunotherapeutic drugs due to less side effects inherent in monosaccharide-containing muramyl peptides (fever, aches, joint pain), and a wide range of therapeutic efficacy. In **Figure 3** shows a graph is shown of the indices of stimulation of the humoral response to ovalbumin in BALB/C mice after intraperitoneal administration of MDP and GMDP in the range from 5x10^-7^ to 5x10^2^ mg/kg (6 animals per dose), while GMDP exhibits more pronounced adjuvant activity than MDP at concentrations of 5 and 5x10^-4^ mg/kg (79).

The first registered drug in the series of disaccharide-containing muramyl peptides is the drug **Licopid^®^** (glucosaminylmuramyl dipeptide, GMDP, glycopin), which has been used since 1996 in the complex therapy of diseases accompanied by secondary immunodeficiency states: chronic, recurrent infections of the upper and lower respiratory tract in the stage of acute and chronic purulent-inflammatory diseases of the skin and soft tissues (pyoderma, furunculosis and others); herpes infection (including ophthalmic herpes); psoriasis (including psoriatic arthritis); pulmonary tuberculosis.

The introduction of the drug licopid into medical practice was preceded by numerous preclinical and clinical studies in Australia (112), Great Britain (113–115) and Russia (116, 117), during which a wide spectrum of biological activity of glucosaminylmuramyldipeptide was revealed. GMDP stimulates development of both cellular and humoral immune responses. When mice are immunized with BCG vaccine, GMDP increases the synthesis of a factor that inhibits the migration of macrophages by 3 times and increases antibody production to corpuscular and soluble antigens by 2–5 times (118). In experimental studies, a pronounced stimulating effect was revealed when using GMDP as an adjuvant in an HIV vaccine containing a recombinant gp120 antigen (107), during *in vitro* immunization to obtain monoclonal antibodies (119), as well as in a DNA vaccine against herpes simplex virus (120) and in acellular pertussis vaccine (121). These studies indicate that GMDP is a promising adjuvant suitable for use in a wide range of vaccines and experiments (122). In addition, the use of GMDP 1-4 days before the introduction of a lethal dose of *E. coli* (123), *Ps. aeruginosa* or LPS protects 60 to 100% of animals from death. GMDP also has antiviral activity: it suppresses the spread of the influenza virus in the body of infected mice (118). GMDP stimulates leukopoiesis: its daily administration to irradiated animals at a dose of 100 μg for 8 days significantly increases both the total number of leukocytes and the number of neutrophils on the 6-7th day after irradiation (124), corrects cytopenia during antiviral therapy for hepatitis C (46). GMDP has antitumor activity: it inhibits the growth of some transplanted tumors, exhibits synergism with other antitumor agents and immunostimulants (125). Thus, a single injection of cyclophosphomide 1 hour before the administration of GMDP significantly inhibits the growth of LLC Lewis carcinoma, and the combination of LPS and GMDP completely cured EL-4 thymoma and MS-11 sarcoma. At the same time, the cured mice developed antitumor immunity (126). The results of preclinical experiments showed the high potential of glucosaminylmuramyldipeptide as an immunotherapeutic agent and formed the basis of clinical trials.

**Clinical trials** of the drug licopid took place in Australia (UNSW Department of Surgery and Department of Oncology, The St. George Hospital), Great Britain (Toxicol Laboratories Ltd, The Royal Masonic Hospital) and in the leading medical institutions in Russia and the CIS countries in accordance with international rules of proper clinical practice (Good Clinical Practice, GCP) using randomized double control (112–117). The drug licopid is widely used in Russia and CIS countries, every person can buy it without prescription (in dose 1 mg per tablets). Analysis of the accumulated material has made it possible to identify several mechanisms underlying the clinical efficacy of the drug. Undoubtedly, the main mechanism of the immunotherapeutic action of the drug licopid is the activation under its influence of anti-infectious protection, cellular and humoral immunity, which is suppressed by secondary immunodeficiencies. Another mechanism includes a pathogenetic effect on the mechanism of allergic inflammation, switching the immune response from Th2-type to Th1, significantly reducing the level of the main markers of atopy IgE and IL-4. In addition, by stimulating the expression of colony-stimulating factors, GMDP corrects cytopenia by accelerating the process of bone marrow hematopoiesis.

The effectiveness of GMDP in the treatment of secondary immunodeficiency states caused by chronic infections of various etiologies has been repeatedly confirmed in clinical practice.

The inclusion of GMDP in the complex therapy of chronic recurrent upper respiratory tract infections in children and adults reduces the number of relapses, the severity and duration of diseases, improvements in the parameters of humoral and cellular immunity. In a pharmaco-epidemiological study to evaluate the effectiveness of the drug licopid in reducing the incidence of frequently and long-term ill children of the Vladimir region, attending closed children’s groups, 214 children with frequent diseases of the respiratory tract (more than 4 times a year) in the remission phase received a course of licopid (156 children aged 1.5 to 6 years, 1 mg per day for 10 days, 58 children aged 7 to 12 years, 2 mg per day for 10 days). Observation of children for one year revealed a decrease in the incidence of acute respiratory infections by 2.24 times (p <0.01) in the group of younger children and by 2.34 times in the group of older children (p <0.01) who received licopid, against the background practically unchanged weighted average indicators of morbidity in children during the study period, who did not receive licopid (127). Comparison of the efficacy of the two regimens of licopid in children with recurrent respiratory tract infections was the subject of the following study (128). In two stages of the study, a total of 90 children (30 in groups) from 2 to 15 years old were included with a frequency of airway respiratory infection (ARI) more than 6 episodes per year and at least 4 over the last six months, with a disease duration of at least 30 days. When prescribing the drug licopid to 30 children without exacerbation in standard age dosages (1 mg per day for a course of 10 days; Scheme No. 1) led to a decrease in morbidity in 60% of children in the study group. 10% of children who received licopid achieved good and 50%- satisfactory efficacy, i.e. there was a decrease in the number of acute respiratory infections by 25-50% (satisfactory effect) and more than a half (achieved the good effect) during the next 6 months after the end of the course of immunomodulatory therapy. Clinical efficacy correlated with an improvement in immunological parameters, which is clearly seen in children with an initially reduced number of CD4+ cells (14 out of 30 children) - an increase in the relative and absolute number of CD4+ cells, immunoregulatory index (IRI), the index of induced chemiluminescence, IgA and IgG levels (p<0.05). The clinical and immunological effect with this dosing regimen persisted for 3 months, which made it possible to determine the interval between the courses of 3 months. The best results in reducing the incidence were obtained by prolonging the prescription of the drug licopid up to 3 months (scheme No. 2) - 1 mg per day, every first 10 days of the month (the interval between the courses of administration was 20 days). A decrease in the number of episodes of respiratory infections was noted in 27 (90%) patients. Of these, half of the children from the study group (15 people) had a satisfactory effect (reduction of ARI episodes by 25-50%), 40% (12 people) had a good effect (reduction of ARI episodes by more than 50%). On the part of immunological parameters, an increase in the level of IgA in saliva and IgG in the blood was noted in the group (p <0.05). When assessing the immunomodulatory effect of licopid in patients with an initial decrease in CD4 + cells, there was a significant increase in the content of serum IgG and IgA, as well as IgA in saliva, IRI, spontaneous and induced chemiluminescence. The preservation of dynamics of immunological and clinical indicators for 6 months with the 2nd scheme of taking the drug licopid made it possible to recommend this particular time interval between the courses. Thus, when prescribing the drug licopid for a longer course (scheme No. 2), better immunological and clinical efficacy was obtained than when prescribing it for a short course (scheme No. 1) or in the comparison group that had not received any licopid (χ^2^> 3.8; p < 0.05).

Analysis of the effectiveness of GMDP in 123 patients with chronic bronchitis in remission (age 43.03 ± 1.4 years, with duration of the disease on average 8.2 ± 1.4 years) revealed the optimal dosage - 2 mg 3 times a day, with which there was no cough in 75% of patients (if present in 100% before treatment); absence of wheezing in the lungs in 76% (if present in 100% before treatment); no shortness of breath in 84% (against 25% value before treatment) (129).

In the treatment of chronic recurrent infections caused by the herpes simplex virus types 1 and 2, taking licopid in combination with antiviral drugs leads to the absence of rashes on the 5-7th day of therapy. At the same time, a pronounced anti-relapse effect was noted - the absence of relapses within 1.5 years of observation in 100% of patients in the group, while in patients of the comparison group, relapses of herpes infection were observed every 3-4 months (130). Such clinical improvement was clearly correlated with improvements in immunity indicators. In the group of patients who received complex therapy with the drug licopid, there was a normalization of the level of the cellular indicators (the number of CD3, CD4 cells) and the functional activity of natural killer cells. When treating cytomegalovirus infection with the drug licopid in the stage of exacerbation, already after 2 weeks from the start of treatment, patients noted a significant improvement in well-being (general weakness, increased fatigue and sweating disappeared), and their temperature also returned to normal. Long-term results - during follow-up for 1 year, there were no acute respiratory infections (ARI) compared to the period before treatment - frequent ARI during the year.

In a double-blind, placebo-controlled study in the treatment of 100 women with local and extensive forms of cervical condylomatosis, the use of licopid at a dosage of 20 mg for 10 days 7 days after laser treatment increased the effectiveness of complex therapy: it reduced the number of relapses by 4 times (131). Treatment with licopid in 207 patients with papillomavirus infection (PVI) of the genitals in a course dose of 200 mg before or after laser destruction was highly effective in 97.6% of cases. Recurrences of PVI were observed only in 2.4% of women after 1 year (132). The efficacy of GMDP monotherapy was also confirmed at a dosage of 10 mg within 10 days after destruction (133).

In the treatment of chronic pyoinflammatory diseases of the skin and soft tissues, the dynamics of 178 patients with furunculosis was analyzed, including 50 patients with acute furunculosis and 128 patients with chronic recurrent furunculosis at the age of 15 to 50 years. GMDP was used in various dosages - 6 mg and 10 mg per day, in combination with etiotropic antibacterial therapy and antistaphylococcal immunoglobulin. When using GMDP at a dosage of 6 mg per day, a positive effect (no relapse of the disease after the end of therapy within 6 months) was noted in 96% of patients. At the same time, in 1 patient with chronic furunculosis against the background of diabetes mellitus, complete recovery was not achieved, but during the course of the disease, positive dynamics were observed (rare relapses with a mild and short-term form). When prescribing GMDP at 10 mg 1 time per day for 10 days, patients with chronic sluggish furunculosis (disease more than 4 years) experienced clinical remission in 97% of cases. It is important to note that the combination of GMDP and antistaphylococcal immunoglobulin in patients with identified antibiotic-resistant staphylococcal strains made it possible to achieve the onset of remission in 99% of patients, whereas when using only antistaphylococcal immunoglobulin, remission was achieved in 40% of cases within six months (134).

In the treatment of chronic purulent-inflammatory diseases of the skin and soft tissues, GMDP was prescribed to children aged 1 to 14 years by mouth 1 mg 1 time per day for 10 days with basic and antibacterial therapy. All 30 patients included in the study suffered from recurrent purulent skin infections for six months or more. The tolerability of the drug licopid was good; during the course of treatment, there were no side effects. At the end of treatment, 27 children (90%) with recurrent purulent skin infections showed a good effect (a decrease in the number of disease relapses by more than 50% within 6 months after the end of treatment compared to the same indicator before treatment). Of these, 20 children (66%) achieved complete remission - there was not a single relapse during the follow-up for six months. After the course of immunotherapy with licopid, the need for prescribing antibacterial agents decreased. Negative dynamics was not observed in any of the patients; a significant increase in the initially decreased relative and absolute values ​​of CD4- lymphocytes and serum IgG content was recorded (135).

The efficacy of GMDP in psoriasis was shown in studies in the UK and Russia (115, 136). Taking 10 mg of GMDP for 10 days during the period of remission helped to reduce the severity of exacerbations and lengthen the period of remission.

Thus, the introduction of the drug GMDP into tactical regimens for the treatment of many diseases, including chronic recurrent bacterial and viral infections, helps to reduce the duration of the disease, increase the effectiveness of standard therapy, and reduce the number of relapses.

Against the background of taking the drug licopid, there is an improvement in the course of not only infectious, but also allergic diseases, both due to the normalization of immunological indicators and the prevention of infectious complications of the underlying disease as a result, and due to the restoration of the balance between Th1 and Th2. In total, 406 patients participated in controlled studies to study the effectiveness of the drug licopid in the treatment of atopic and infectious-allergic diseases, including 140 adults and 266 children (excluding the comparison group). In a clinical study of the effect of the drug licopid on the course of IgE-mediated atopic dermatitis (AD) of moderate severity in the acute stage, 46 children aged 6 to 9 years took licopid sublingually 30 minutes before meals, 1 mg 2 times a day for 5 days; then - 1 mg once a day for 15 days (course dose - 25 mg). It was noted that the inclusion of the drug licopid in complex therapy contributed to a 3.5-fold reduction in the area of ​​the lesion (with traditional therapy - 1.8 times) after 1 month, and after 2 months by another 3 times, while in 40% of patients skin manifestations completely disappeared. Analysis of the intensity of clinical manifestations revealed a significant decrease by 4 times after a month from the start of treatment and 8 times after 2 months. Evaluation of subjective sensations showed a faster improvement in the use of combined immunotherapy. Thus, in 26 patients of this group, skin itching and sleep disturbance were absent by the end of the 1st month of treatment, only in 5 patients there was a slight itching. The study of the content of total serum IgE after treatment revealed a significant decrease only in children who received licopid. Thus, a clinical and experimental study revealed a significant effect of the drug licopid on the course of blood pressure in children, which can significantly improve the quality of life of patients (137).

Similar data were obtained when studying the effectiveness of GMDP in the complex therapy of severe atopic dermatitis in adults. The study included 72 patients aged 17 to 60 years, 44 of whom received GMDP, 1 mg per day (21 people) and 10 mg per day (23 people) for 10 days. The most common concomitant pathology was diseases with an allergic component (37.5%); taking into account bronchial asthma, these patients accounted for 48.6% of all observed patients. Before treatment, all patients were bothered by itching of different degrees of intensity. All patients showed positive dynamics of clinical manifestations, but reliably significant results were observed only in the groups of patients who received GMDP. So, after using the drug licopid 10 mg, the SCORAD index (IS) decreased from 58.0 ± 3.96 to 17.2 ± 2.2 (p <0.05). Therapy with the drug licopid 1 mg led to a decrease in IS from 47.9 ± 3.7 to 10.5 ± 1.4 (p <0.05), while after traditional therapy the index decreased only to 25.1 ± 2.5, with its initial value 55.4 ± 3.9 (p <0.05). A correction of laboratory and immunological parameters occurred only in the group of complex treatment with the use of the drug licopid 10 mg. This group of patients showed a significant decrease in spontaneous chemiluminescence (1.23 ± 0.04 before treatment, 1.1 ± 0.03 - after treatment, p <0.05) and stimulated chemiluminescence of neutrophils (1.65 ± 0.1 - before treatment and 3.0 ± 0.5 - after treatment, p <0.05). There was also a decrease in the initially increased number of neutrophils (4.0 ± 0.24 before and 3.1 ± 1.2 in 1 μl of blood plasma after treatment, p <0.05) and leukocytes (7.4 ± 0.5 before and 5.8 ± 0.3 in 1 μl of blood plasma after treatment, p <0.05). When studying the long-term results, the follow-up period ranged from 6 months to 1.5 years. Anamnestic data was taken into account, including the frequency, duration of exacerbations, the severity of the clinical picture, the intensity of itching, changes in the nature of the course of concomitant diseases. The long-term results were studied in 60 patients with atopic dermatitis, of whom 19 - after using the drug licopid 10 mg, in 18 - using the drug licopid 1 mg, in 23 - who received only traditional therapy. A decrease in the frequency of exacerbation was noted by 16 patients out of 19 (84%) after complex treatment using the drug licopid 10 mg, in 61.1% of patients receiving licopid 1 mg (11 patients out of 18), and in 52% (12 out of 23 patients) who received traditional therapy. A statistical assessment of the frequency of exacerbations of atopic dermatitis indicates a significant positive dynamics in patients after complex therapy with the inclusion of the drug licopid compared with the group of patients receiving traditional therapy (χ^2^ = 4.8; p <0.05) (138).

The inclusion of GMDP (10 mg once a day, 10 days) in a complex with allergen-specific immunotherapy (ASIT) in 128 patients with infectious-allergic diseases of the respiratory tract and skin, along with an increase in clinical efficacy, made it possible to halve the number of adverse reactions caused by the main treatment, the number and intensity of skin tests with specific allergens. The study of the immune status of patients showed a more significant effect of combined immunotherapy on the system of neutrophilic phagocytosis, quantitative and functional parameters of the cellular link of immunity and the level of total IgE (139–141).

Advanced study of the possibilities of immunotropic therapy using the drug licopid in patients with bronchial asthma (BA) has demonstrated the effect of the drug both on the clinical manifestations of the disease (142, 143) and on its pathogenetic mechanisms - the balance of Th1/Th2 lymphocytes and the level of the main markers of atopy - IL-4, IgE (144). Thus, the previously obtained clinical data indicating an improvement in the condition of patients with atopic diseases during therapy with licopid can be interpreted not only as the prevention of infectious complications of the underlying disease, but also as the pathogenetic effect of the drug on the process of allergic inflammation - restoration of the balance of Th1/Th2 lymphocytes.

The antitumor activity of GMDP was studied in Russia and Australia for various oncological pathologies in a total of 620 patients (excluding patients from the comparison group). In a clinical study of the efficacy of GMDP in the treatment of 164 patients with a widespread tumor process of the gastrointestinal tract (68.7% of patients with stage IV of the disease), the immunotherapeutic activity was assessed when the drug was prescribed in two schemes (145). One course of GMDP, 10 mg once a day, for 7 days, course dose - 70 mg, was received by 86 patients in combination with basic symptomatic therapy (first group). Two courses of the GMDP 10 mg once a day for 7 days with an interval of two weeks between courses, a course dose of 140 mg was received by 78 patients in combination with basic symptomatic therapy (second group). The comparison group consisted of 82 patients who received basic symptomatic therapy. The effectiveness of therapy was assessed by comparative analysis of clinical and immunological parameters in each group at the following stages: initial (before the start of treatment); on the 21st day from the start of treatment (2 weeks after the end of the first course of treatment with licopid); on the 42nd day from the start of treatment (2 weeks after the second course of treatment with GMDP); on the 100th day from the start of treatment (2 months after the end of the second course of treatment with GMDP). In the immunological indicators of patients in groups 1 and 2, there were positive trends in the form of an increase in the number of immunocompetent cells (CD3, CD4), immunoregulatory index (IRI), an increase in the phagocytic activity of neutrophils, but the immunogram indicators remained low and did not reach the lower limit of the norm. Analysis of the results on the 42nd day of the study showed that the patients of the 2nd group (who received two courses of GMDP according to the protocol) showed significantly significant positive changes in the T-cell and phagocytic links, the indicators of which were close to normal values. In addition, there was a normalization of the level of NK cells, IgA, a decrease in the CEC. Patients of group 1 (who received one course of the drug licopid) retained minor positive changes. At the same time, the patients of the comparison group showed a tendency to aggravate immunosuppression from the T-cell and phagocytic links. The study of the immune profile on the 100th day from the start of treatment revealed a tendency towards increased immunosuppression in the comparison group and in the 1st group of patients who received one course of immunotherapy. The immunological parameters of the patients in the 2nd group (two courses of immunotherapy) remained within the lower limits of normal values. The results obtained from large-scale studies with common tumor processes indicate the advisability of including GMDP in a complex of treatment and rehabilitation measures in order to correct immunological parameters and adequate control of the infectious syndrome according to the scheme: 10 mg 1 time per day, daily for 7-10 days, repetition course in 2-4 weeks.

Data on the study of the biological activity and clinical efficacy of GMDP indicates its ability to regulate the function of all major populations of immunocompetent cells: monocytes, macrophages, T- and B-lymphocytes, neutrophils, natural killers; correct Th1/Th2 imbalance, ensuring the effective functioning of the immune system. The pharmacological activity of the drug is done through the binding of its active unit, GMDP, with the intracellular receptor of innate immunity NOD2. The binding of GMDP to the receptor leads to a whole series of events inside the cell, which ends with the activation of the nuclear factor NF-kB and the production of a number of key cytokines - IL-1, IL-6, IL-12, TNF-α, interferon-gamma, colony-stimulating factors. In addition to cytokines, the activation of NF-kB increases the expression of adhesion molecules, acute phase proteins, inflammatory enzymes (NO-synthase and cyclooxygenase), MHC molecules, defensins, etc. (146–149). At the level of the organism, the activation of the synthesis and secretion of the listed molecules leads to the development of an inflammatory reaction with the activation of all available systems of protection against pathogens. The activation of innate immunity in persons with primary immunodeficiencies, for example, with agammaglobulinemia, is the main way to protect against infection (150).

GMDP is involved not only in ensuring the immune homeostasis of the human body (151, 152), but also in regulating the composition and diversity of representatives of the microbiological community of the oral mucosa (153), and it also increases the effectiveness of anti- *Helicobacter pylori* therapy in the stomach (154).

GMDP-A, a derivative of GMDP (155, 156), has demonstrated high efficacy as a part of complex anticancer therapy and is currently ready for clinical trials. The inclusion of GMDP-A in the treatment of transplantable tumors Lymphocytic leukemia P388 and Melanoma B16 reduces the frequency of metastasis and inhibits tumor growth in 95% (P388) and in 100% (B16) cases, increases the life span of animals and reduces the amount of cytostatic cisplatin by 30%.

Acting on the key molecular target in the immune system, the NOD2 receptor, the GMDP mimics the process of detecting peptidoglycan fragments in bacteria. Thus, the effect of the drug is as close as possible to the process of natural immunoregulation, which is done in the body under the influence of bacteria. Glucosaminylmuramyldipeptide is included in the composition of several dozen patents, most of which are registered by Curevac Ag.

The liposomal drug form of N-acetylglucosamine-N-acetyl-muramyl-L-alanyl-D-isoglutamine-L-alanyl-glyceryl dipalmitate (**ImmTher**) has been declared orphan by the Food and Drug Administration (FDA). The main indications for its appointment are osteosarcomas, Ewing’s sarcomas and colorectal adenocarcinoma with metastases to the lungs and liver. In clinical trials, multiple injections of the drug with concomitant radio- and chemotherapy caused leukocytosis due to neutrophils and a significant increase in tumor necrosis factor (TNF). ImmTher induces the expression and production of interleukin IL-1beta, -1alpha, IL-6, IL-8, IL-12, tumor necrosis factor alpha, but not IL-2 or IL-10. At the same time, cytotoxic activity was recorded against osteosarcoma, Ewing’s sarcoma, melanoma, but not breast cancer (157, 158). In January 2020, the 2nd phase of the clinical trial ended ahead of schedule due to the lack of a sufficient number of patients required for statistical significance (159).

ImmTher is part of the composition of about two hundred patents, most of which are registered by Curevac Ag.

**Liasten** -** **a disaccharide-containing muramyl pentapeptide isolated from *Lactobacillus Delbrueckii* - is produced since 2000 in the form of a powder for the solution for injections, containing 0.002 g of the active substance, as well as in the form of 2 mg tablets. Liasten is used in the treatment of diseases accompanied by secondary immunodeficiency and leukopenia, in acute and chronic radiation injuries, in the surgical treatment of oncological diseases, in acute and chronic bacterial infections. The course of treatment requires 3-5 subcutaneous or intramuscular injections at a daily dose of 2 mg with an interval of 5-7 days between them. Side effects can manifest in the form of local reactions at the injection site, hyperthermia and joint pain. During clinical trials on 81 patients with breast cancer (BC), when assessing the effectiveness of the drug, the subjective feelings of patients who underwent chemotherapy and radiation therapy, as well as changes in blood leukogram indicators (before the start of taking the drug 3.0 ± 0.3 * 10^9^/l, during treatment 3.7 ± 0.4*10^9^/l, after the end of the drug intake 3.8 + 0.6*10^9^/l) (160). The combined treatment of patients with stage I-IV breast cancer in combination with three-, five- fold subcutaneous administration of an immunomodulator as an accompaniment drug prevented the development of leukopenia throughout the entire course of chemotherapy and radiation therapy. In leukopenia that developed as a result of treatment of breast cancer patients, the drug restored the number of leukocytes, which made it possible to complete the course of chemotherapy and/or radiation therapy. When assessing the 5-year survival rate of patients with stage II-III breast cancer, in whom complex treatment an immunomodulator from *Lactobacillus Delbrueckii* was used, an increase in overall and disease-free survival was found in comparison with the data in the control group of patients by 13 and 10%, respectively (161). Clinical trials of the effectiveness of the drug in the complex treatment of 33 patients with acute and chronic inflammatory diseases of the bronchopulmonary system in the acute phase showed good tolerability of the drug (temperature reactions in 2, moderate local manifestations also in 2 out of 33 patients), a reduction in the course of antibiotic therapy, and time spent in bed for 1.5-2 days, reducing the number of exacerbations. Researchers explained the effectiveness of the drug by nonspecific stimulation of the cellular link of immunity and the phagocytic system by increasing the production of IL-1 (162). Stimulation of the cellular and humoral link of immunity also explains the positive effect of Liasten in the complex treatment of patients with severe burns and newly diagnosed destructive pulmonary tuberculosis (163, 164).

The drug **Polimuramil** is produced in the form of a solution for intramuscular administration since 2013 and is a complex of muramyl peptides, consisting of three main components: β-N-acetyl-D-glucosaminyl-(1→4)-N-acetyl-D-muramoyl-L-alanyl-D-isoglutaminyl-meso-diaminopimelic acid (HMtri), β-N-acetyl-D-glucosaminyl-(1→4) -N-acetyl-D-muramoyl-L-alanyl-D-isoglutaminyl-meso-diaminopimeloyl -D-alanine (HMtetra) and the dimer of HMtetra (diHMtetra), in which the monomeric residues of HMtetra are connected by an amide bond between the carboxyl group of the terminal D-alanine of one residue of HMtetra and the ω-amino group of meso-diaminopimelic acid of another residue of HMtetra. The drug is used in adults simultaneously with antibacterial drugs in the complex therapy of secondary immunodeficiency states, manifested in the form of chronic, sluggish, recurrent infectious and inflammatory processes of the skin, soft tissues; acute and chronic pyoderma, osteofolliculitis, sycosis, deep folliculitis, carbuncle, hidradenitis, furunculosis, abscesses, impetigo; dermatoses complicated by secondary infection (atopic dermatitis, eczema); for the treatment and prevention of surgical infections, including postoperative purulent-septic complications. During the first phase of clinical trials, the drug showed good tolerance and safety at a dose of up to 400 μg with a single administration and up to 1 mg with a course administration. Polimuramil increased the ability of blood leukocytes to produce reactive oxygen species and kill S. aureus, caused an increase in serum levels of C-reactive protein, as well as cyto- and chemokines involved in the innate immune response (MIP-1β, MCP-1, IL-6) (165). During subsequent placebo-controlled clinical trials of phase II/III in patients with purulent surgical infection (n = 30 people), daily administration of PM at a dose of 200 μg intramuscularly for 5 days helped to normalize body temperature, reduce the severity of pain and general discomfort. The components of the drug are ligands for the innate immunity receptors NOD1 and NOD2 (166). According to laboratory studies, the drug stimulated components of the immune system necessary to combat the extracellular forms of bacteria: increased serum IgM levels, increased intracellular bactericidal activity of leukocytes, and phagocytic index of neutrophils (167). Polimuramil is mentioned in two patents of the Corus Pharm company.

Experimental studies and clinical experience in the use of drugs based on muramyl peptides, acting on innate immunity receptors and modulating the production of key cytokines, indicate the possibility of influencing the onset and course of the infectious process caused by various pathogens, potentiating the effect of anticancer drugs, regulating the ratio of populations of immunocompetent cells, affecting the pathogenesis of the disease. In modern conditions, when an increasing number of microorganisms resistant to all known antibiotics threatens the existing control of infectious processes, and conventional surgical intervention can become fatal, immunomodulators based on muramyl peptides acquire a major advantage in comparison with other drugs: by acting not on the pathogen, but on the immunocompetent cells of the macro organism, they activate a cascade of immune responses aimed at protecting against the pathogen.

## Conclusion

1. The possibility of chemical modification of muramyl peptides presents great prospects for obtaining new drugs that are effective both in the fight against pathogens and in the correction of immunity dysfunctions arising from allergic, oncological and other pathological processes, which is proved by the inclusion of muramyl peptides in several thousand patents, registered worldwide. The most suitable analogs are disaccharide-containing derivatives that do not have a pyrogenic effect.
2. Muramyl peptides, as fragments of bacterial cell walls, are important model compounds for determining the mechanisms of realization of innate and acquired immunity, with their help NLR receptors were characterized and the role of these receptors in the pathogenesis of diseases was determined. In this regard, increased attention was drawn to analogs of muramyl peptides, which can be purposefully used to obtain a reliable, well-predictable effect, which is extremely important in the creation of drugs and effective vaccines.
3. Representatives of the microbiological community respond to muramyl peptides by modifying life cycles of development and diversity.
4. Muramyl peptides affect all populations of immune cells, taking part in the correction of pathological processes in primary and secondary immunodeficiency, chronic infectious diseases, oncological and allergic processes, vaccination. They are responsible for maintaining “trained immunity” capable of quickly and adequately responding to the pathogen in order to ensure homeostasis, which forms the basis to use drugs based on muramyl peptides for the prevention of infectious diseases.

## Author Contributions

All authors listed have made a substantial, direct and intellectual contribution to the work, and approved it for publication.

## Conflict of Interest

The authors declare that the research was conducted in the absence of any commercial or financial relationships that could be construed as a potential conflict of interest.
